# Shared mechanisms linking PM_2.5_ exposure, obstructive sleep apnea and obesity

**DOI:** 10.3389/frsle.2026.1918405

**Published:** 2026-08-14

**Authors:** Bianca C. Schimenes, Tathiana A. Alvarenga, Sergio B. Tufik, Sergio Tufik, Monica L. Andersen

**Affiliations:** 1 Departamento de Psicobiologia, Universidade Federal de São Paulo (UNIFESP), São Paulo, Brazil; 2 Instituto do Sono, Associação Fundo de Incentivo à Pesquisa (AFIP), São Paulo, Brazil

**Keywords:** sleep, air pollution, environmental health, environmental pollution, fine particulate matter, metabolic health, obesity, obstructive sleep apnea

## Abstract

Obesity and obstructive sleep apnea (OSA) are highly prevalent and interconnected conditions that contribute to the global burden of chronic non-communicable diseases. Fine particulate matter (PM_2.5_), a major component of air pollution, has emerged as a relevant factor linked to metabolic dysfunction and sleep disturbances. Chronic exposure to PM_2.5_ has been related to higher body mass index (BMI), central fat deposition, metabolic syndrome, and increased OSA risk and severity. Obesity remains the main modifiable risk factor for OSA, whereas intermittent hypoxia and sleep fragmentation can aggravate hormonal, inflammatory, and metabolic disturbances involved in weight gain. PM_2.5_ exposure and OSA overlap through pathways involving systemic inflammation, oxidative stress, neuroendocrine dysregulation, and gut microbiota alterations. These converging mechanisms highlight the potential role of environmental pollution and sleep disruption in obesity pathophysiology, particularly in urban populations chronically exposed to adverse environmental conditions. This review synthesizes current epidemiological and mechanistic evidence, identifies important knowledge gaps, and proposes shared biological pathways that may inform future research on the interplay among PM_2.5_ exposure, OSA, and obesity.

## Graphical Abstract

**Figure:**
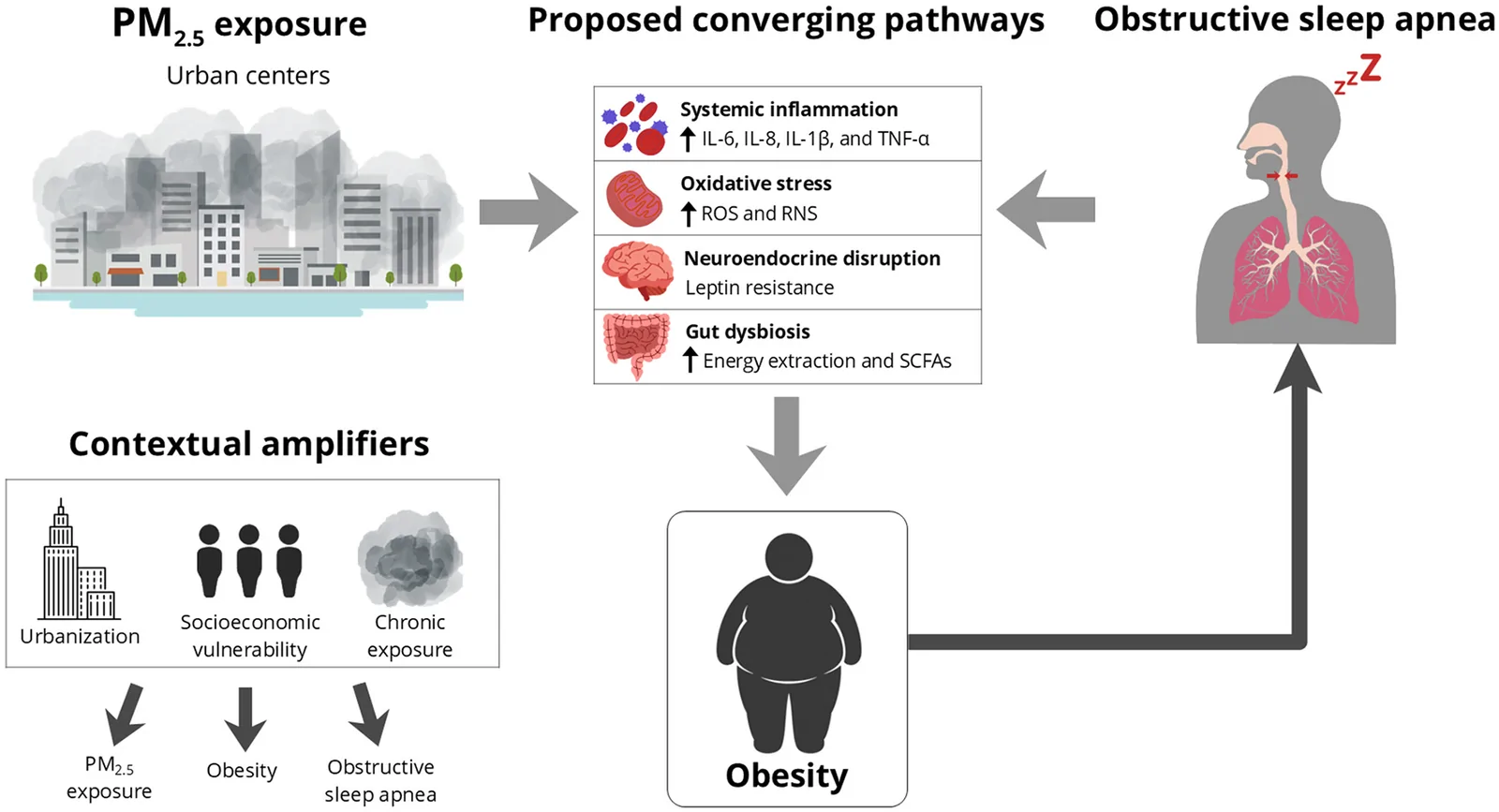
Proposed mechanisms linking fine particulate matter (PM_2.5_) exposure and obstructive sleep apnea (OSA) to obesity. Shared pathways include systemic inflammation, oxidative stress, neuroendocrine disruption, and gut dysbiosis, which may promote metabolic dysfunction and adiposity accumulation, particularly in urban populations exposed to chronic environmental stressors. Image created with Canva.

## Introduction

The global rise in chronic non-communicable diseases has become one of the most pressing public health challenges of modern society. Obesity, diabetes *mellitus*, neoplasms, chronic respiratory conditions, and cardiovascular diseases now dominate the worldwide health burden, reflecting complex interactions between behavioral, biological, and environmental factors (Freihat et al., 2025; Koliaki et al., 2023). Among these contributors, air pollution has emerged as a major global health threat, accounting for approximately 9 million premature deaths annually, and increasing 66% from 2000 to 2015 (Fuller et al., 2022).

Obesity stands out as a critical driver of disease due to its widespread prevalence and close connection to multiple metabolic and physiological disturbances, including sleep disorders (Figorilli et al., 2025). In parallel with the escalating obesity epidemic, sleep disturbances have become increasingly prevalent, with obstructive sleep apnea (OSA) being one of the most common yet underrecognized disorders (Tufik et al., 2026). Affecting more than 37% of adults in some populations (Tufik et al., 2026), OSA is strongly influenced by excess body fat and represents an important, but often overlooked consequence of the broader metabolic problem associated with obesity (Figorilli et al., 2025).

The relationship between obesity and sleep is inherently bidirectional. Excess adiposity influences upper airway collapsibility through fat deposition in the cervical region, augmented tongue size, and decreased lung volume, predisposing individuals to OSA (Figorilli et al., 2025). In turn, sleep disturbances, including sleep fragmentation and deprivation, promote weight gain via alterations in appetite-regulating hormones, elevated caloric intake, lower levels of physical activity, and systemic inflammation (Figorilli et al., 2025; Zhu and Lv, 2026).

Although lifestyle determinants, including diet, physical inactivity, and socioeconomic factors, play a major role in the development of these conditions, increasing attention has been directed toward environmental exposures as complementary contributors. Among these, air pollution, particularly fine particulate matter (PM_2.5_), has emerged as a relevant element, especially in densely populated urban environments where exposure levels remain high (Wu et al., 2024).

Accumulating epidemiological data has linked PM_2.5_ exposure not only to cardiopulmonary disease (Sangkham et al., 2024), but also to obesity-related indicators, including higher body mass index (BMI), systemic and abdominal adiposity, and metabolic dysfunction (Wu et al., 2024; Yuan et al., 2026). Simultaneously, air pollution has been related to poorer sleep health (Liu et al., 2020), raising the possibility that environmental conditions may impact both metabolism and sleep.

Emerging findings suggest that PM_2.5_ exposure and OSA might share overlapping pathways that contribute to obesity development (Lee, 2025). This possibility is markedly concerning in large urban centers, where high levels of air pollution coexist with substantial obesity rates and a rising prevalence of OSA (Billings et al., 2019; He et al., 2026; Luo et al., 2024; Munir et al., 2026), potentially compounding metabolic risk at the population level. Although PM_2.5_, OSA, and obesity have each been extensively investigated, these conditions are often examined in isolation. Less attention has been given to the possibility that air pollution and OSA could converge through shared biological processes that promote obesity development.

This review summarizes current evidence on the associations between PM_2.5_, OSA, and obesity with particular emphasis on shared mechanisms through which air pollution and OSA may promote obesity development.

## Literature search strategy

This article was designed as a narrative review aiming to summarize current evidence regarding the relationships among PM_2.5_ exposure, OSA, and obesity, with emphasis on shared biological mechanisms involved in obesity pathophysiology.

A literature search was conducted in PubMed and Google Scholar for articles published up to June 2026, with priority given to studies published from January 2000 onward. Search terms included combinations of the following keywords: (“air pollution” OR “PM_2.5_” OR “fine particulate matter” OR “particulate matter”) AND (“obesity” OR “adiposity” OR “metabolic dysfunction” OR “metabolic health”) AND (“obstructive sleep apnea” OR “sleep apnea”) together with mechanism-related terms including “inflammation”, “oxidative stress”, “gut microbiota”, “gut dysbiosis”, “lipopolysaccharides”, “leptin resistance”, and “neuroendocrine regulation”. Only articles published in English were considered.

Epidemiological, clinical, experimental, mechanistic, and review articles were considered according to their relevance to the topic. Additional studies were identified through manual screening of the reference lists of selected publications. Preference was given to recent systematic reviews, meta-analyses, large population-based studies, and mechanistic investigations that contributed to understanding the biological links between PM_2.5_ exposure, OSA, and obesity.

As a narrative review, this article did not follow a predefined systematic review protocol, formal study selection process, or methodological quality assessment. Consequently, the synthesis reflects the authors' interpretation of the available literature and may be subject to selection bias.

## Fine particulate matter: an overview

Beyond its well-established respiratory and cardiovascular consequences (Sangkham et al., 2024), air pollution has been recently related to metabolic and sleep conditions (Yuan et al., 2026; Liu et al., 2020).

Among the different particulate matter (PM) fractions, PM_2.5_, defined as particles with an aerodynamic diameter of ≤ 2.5 μm, is of particular concern because of its ability to penetrate deeply into the respiratory tract, reach the alveoli, and influence multiple biological systems involved in metabolic regulation. PM_2.5_ comprises a heterogeneous mixture whose composition and toxicity vary according to emission source, geographic region, season, and atmospheric conditions (Thangavel et al., 2022). It contains black carbon, polycyclic aromatic hydrocarbons, particle-bound organic compounds, aryl hydrocarbons, heavy metals, inorganic ions, minerals, diverse organic compounds, and biologically active components (Thangavel et al., 2022), including lipopolysaccharides (LPS) (Chen et al., 2021).

Following pulmonary deposition, PM_2.5_ may trigger systemic effects through pulmonary inflammation, oxidative stress, translocation of soluble constituents, and, to a lesser extent, direct particle translocation, thereby contributing to metabolic and inflammatory disturbances beyond the lungs (Thangavel et al., 2022).

A growing body of epidemiological data suggests that chronic exposure to PM_2.5_ contributes to metabolic alterations, including higher BMI, central adiposity, and metabolic syndrome (Wu et al., 2024; Yuan et al., 2026), as well as with a higher prevalence and severity of OSA (Billings et al., 2019; He et al., 2026; World Health Organization, 2021; Pai et al., 2022; Seok and Yoon, 2026).

## Fine particulate matter and obesity

Expanding research has linked PM_2.5_ exposure to adiposity apart from traditional behavioral determinants. An umbrella review integrating multiple systematic reviews and meta-analyses concluded that PM_2.5_ is frequently related to greater obesity risk across diverse populations and age groups (Luo et al., 2024). Complementing these findings, a recent meta-analysis in adults found significant associations between PM_2.5_ and higher BMI, waist circumference, and waist-to-hip ratio, reinforcing links not only with overall body weight, but also with central fat accumulation and cardiovascular risk (Munir et al., 2026).

Early life vulnerability has emerged as a critical concern. In a 14-year longitudinal cohort, incremental rises in PM_2.5_ were related to more than a 5% greater risk of incident childhood overweight or obesity, suggesting that chronic pollutant exposure could influence weight trajectories from early developmental stages onward (Wang et al., 2024). Childhood and adolescence may represent particularly vulnerable developmental windows, as exposure to PM_2.5_ during these periods could have long-lasting effects on metabolic regulation, adiposity trajectories, and sleep health (Santoso et al., 2026). However, longitudinal studies simultaneously evaluating environmental exposure, sleep outcomes, and obesity from early life into adulthood remain scarce.

Recent studies applying causal inference approaches have further strengthened this association by demonstrating that PM_2.5_ can contribute to higher triglycerides, glycated hemoglobin, visceral adipose tissue accumulation, and pancreatic fat deposition (Wu et al., 2024). Additional population-based evidence has related particulate pollution to greater systemic and abdominal obesity (Yuan et al., 2026), consistent with the hypothesis that PM_2.5_ could promote metabolically adverse fat distribution rather than weight gain alone.

Socioeconomic disparities might modify this connection. The relationship between PM_2.5_ and obesity can be influenced by socioeconomic status, with socially vulnerable populations often experiencing disproportionate pollutant burden alongside reduced protective resources, potentially intensifying obesity susceptibility (Peng et al., 2025). This pattern reflects a broader global inequity, as The Lancet Commission on Pollution and Health documented that more than 90% of deaths caused by air pollution occur in low- and middle-income countries, where rapid urbanization, environmental degradation, and constrained healthcare infrastructure frequently coexist (Fuller et al., 2022).

Collectively, these findings support a consistent association between chronic PM_2.5_ exposure and obesity across different populations and life stages. Although the magnitude of the observed associations varies among studies and residual confounding cannot be excluded. The convergence of data from longitudinal cohorts, meta-analyses, and causal inference studies supports the hypothesis that PM_2.5_ contributes to obesity development and adverse metabolic health beyond traditional lifestyle-related risk factors.

## Fine particulate matter and obstructive sleep apnea

In addition to its association with obesity, increasing evidence suggests that PM_2.5_ exposure may also contribute to the development and progression of OSA.

In the Multi-Ethnic Study of Atherosclerosis, each 5 μg/m^3^ higher annual PM_2.5_ concentration was linked to approximately 60% greater odds of moderate-to-severe OSA (Billings et al., 2019). More recently, a systematic review and meta-analysis identified that for every 10 μg/m^3^ rise in PM_2.5_, short-term exposure (from < 24 h to 3 weeks) exhibited a 2.25% higher apnea-hypopnea index (AHI), while long-term exposure (≥1 year) led to a 13.33% higher AHI (He et al., 2026), reinforcing both acute and chronic relationships. These findings are particularly concerning given that the World Health Organization recommends an annual mean PM_2.5_ concentration of no more than 5 μg/m^3^ (World Health Organization, 2021), a threshold exceeded by more than 50% of the global population (Pai et al., 2022). Recent population-based results further indicated that higher 30-day and 1-year ambient PM_2.5_ levels were related to increased OSA risk, supporting the relevance of both intermediate and chronic pollutant burden (Seok and Yoon, 2026).

Not all studies report uniform connections across disease severity. In an urban Taiwanese cohort, PM_2.5_ was not significantly associated with body composition changes or OSA severity in individuals with mild disease (AHI < 15 events/hour), whereas nitrogen dioxide (NO_2_) showed stronger links with lower limb fluid redistribution and mild OSA exacerbation (He et al., 2023).

Vulnerable populations can be affected early in life. In children aged 6 to 12 years, higher indoor PM_2.5_ concentrations elevated the odds of sleep disordered breathing, highlighting the potential influence of particulate pollution in pediatric sleep health (Wang et al., 2025). These studies suggest that PM_2.5_ may represent a meaningful environmental factor related to OSA, with a greater relevance in urban settings where pollutant concentrations regularly exceed recommended limits.

Overall, the available epidemiological evidence supports an association between PM_2.5_ exposure and OSA, although effect sizes vary according to exposure duration, pollutant composition, and study population. Future longitudinal studies using objective exposure assessment and polysomnography are needed to clarify causality and identify the populations most susceptible to the adverse effects of PM_2.5_.

Taken together, these epidemiological findings point toward a potential contribution of PM_2.5_ exposure to both obesity and OSA. However, few studies have simultaneously evaluated these conditions within the same population, making it difficult to determine whether PM_2.5_ acts independently, additively, or synergistically. Addressing this knowledge gap will be essential for understanding the interconnected role of environmental pollution in metabolic and sleep health.

## Obesity in individuals with obstructive sleep apnea

Obesity and OSA are deeply interconnected conditions that mutually reinforce one another. Obesity remains the strongest modifiable risk factor for OSA, with excess adiposity having a substantial impact on disease risk and severity across populations (Martins et al., 2007; Esmaeili et al., 2025; Alenezi et al., 2024). Recent data revealed that OSA is frequently diagnosed in individuals with overweight and obesity, strengthening the overlap between metabolic dysfunction and this respiratory sleep disorder (Esmaeili et al., 2025; Alenezi et al., 2024). In parallel, the São Paulo Epidemiologic Sleep Study (EPISONO) underlined OSA as a major public health concern in urban populations, reporting high prevalence rates and identifying obesity-related measures, specifically BMI, visceral fat accumulation, and neck circumference, among its most important associated factors (Tufik et al., 2026; Martins et al., 2007).

The impact of obesity on OSA development extends beyond body weight alone. Excess fat accumulation in the cervical region, increased tongue volume, and central adiposity can promote upper airway narrowing and collapsibility, predisposing individuals to recurrent nocturnal airway obstruction (Martins et al., 2007). Recent imaging-based investigations highlight the importance of alterations related to obesity in upper airway anatomy as key contributors to OSA pathophysiology (Xu et al., 2025).

While obesity heightens OSA susceptibility, sleep fragmentation, intermittent hypoxia, and reduced daytime functioning, OSA may promote weight gain through adverse metabolic, hormonal, and behavioral changes (Figorilli et al., 2025; Heffernan et al., 2024). Some of these obesity promoting responses, including systemic inflammation, oxidative stress, and metabolic dysregulation, overlap with pathways linked to chronic PM_2.5_ exposure, raising the possibility that air pollution and OSA may interact through shared mechanisms that further amplify obesity risk (Thangavel et al., 2022; Heffernan et al., 2024; Li et al., 2022; Zhang et al., 2024; Kheirandish-Gozal and Gozal, 2019; Furukawa et al., 2004). The epidemiological intersection between PM_2.5_ exposure, OSA, and obesity poses a major question: could these conditions share common biological processes that promote metabolic dysfunction?

Rather than representing isolated processes, PM_2.5_ exposure, OSA, and obesity may interact through reinforcing biological pathways. Obesity increases susceptibility to OSA, whereas intermittent hypoxia and sleep fragmentation aggravate inflammation, oxidative stress, hormonal dysregulation, and metabolic impairment that may further promote weight gain. In parallel, chronic PM_2.5_ exposure activates many of these same pathways. Although direct evidence demonstrating synergistic interactions is still scarce, current findings support the hypothesis that environmental pollution and OSA may jointly contribute to obesity progression through convergent mechanisms.

## Shared biological pathways linking fine particulate matter and obstructive sleep apnea to obesity

The epidemiological associations described above suggest that PM_2.5_ exposure and OSA may converge through common biological pathways involved in obesity pathophysiology. Current data indicates that these interactions involve systemic inflammation, oxidative stress, neuroendocrine dysregulation, and gut microbiota alterations.

### Systemic inflammation and oxidative stress

Once in circulation, PM_2.5_ can trigger systemic inflammation and oxidative stress, characterized by increased expression and release of pro-inflammatory cytokines, including interleukin-6 (IL-6), IL-8, IL-1β, and tumor necrosis factor alpha (TNF-α), alongside enhanced production of reactive oxygen and nitrogen species (ROS and RNS) (Thangavel et al., 2022; Zou et al., 2020). These responses can be mediated by the activation of innate immune signaling cascades, such as toll like receptor 4 (TLR4) and downstream nuclear factor kappa B (NF-κB), which play central roles in linking environmental stimuli to inflammatory and metabolic alterations (Chen et al., 2021; Zhang et al., 2024).

OSA contributes to a persistent pro-inflammatory state, leading some authors to propose that it should be regarded as a low-grade chronic inflammatory disorder. Elevated circulating levels of inflammatory mediators, including IL-6 and TNF-α, are consistently observed in affected individuals (Li et al., 2022). This inflammatory burden is largely driven by recurrent episodes of intermittent hypoxia and reoxygenation during sleep, which promote oxidative stress and activation of inflammatory pathways. Sleep fragmentation and recurrent arousals sustain sympathetic nervous system activation and amplify inflammatory signaling (Kheirandish-Gozal and Gozal, 2019; Gileles-Hillel et al., 2016; Sforza and Roche, 2016; Mesarwi et al., 2015). These alterations lead to systemic oxidative stress and metabolic dysfunction, potentially favoring obesity development and progression (Gileles-Hillel et al., 2016; Mesarwi et al., 2015).

Low-grade systemic inflammation and oxidative stress are central features in obesity pathophysiology (Gregor and Hotamisligil, 2011). Excess adipose tissue is characterized by intensified production of pro-inflammatory markers, including C-reactive protein (CRP), IL-6, and TNF-α, leading to insulin resistance and metabolic dysfunction (Gregor and Hotamisligil, 2011; Ouchi et al., 2011). In parallel, enhanced generation of ROS contributes to mitochondrial impairment and further amplifies inflammatory signaling (Furukawa et al., 2004), creating a self-sustaining cycle of metabolic disruption. These processes not only reflect the expansion of adipose tissue, but also actively influence the development and progression of obesity.

### Neuroendocrine dysregulation and appetite control

Beyond systemic inflammatory responses, experimental evidence indicates that PM_2.5_ exposure might directly influence obesity development through central metabolic dysregulation. In a controlled murine model, chronic exposure to PM_2.5_ led to greater body weight, adiposity, and impaired metabolic homeostasis, even in the absence of dietary changes. These effects were associated with hypothalamic inflammation, characterized by increased expression of pro-inflammatory mediators and activation of TLR4-dependent mechanisms. In the same study, PM_2.5_ exposure disrupted leptin signaling, promoting leptin resistance, a key feature in obesity pathophysiology that impairs appetite regulation and energy balance (Campolim et al., 2020). Hypothalamic inflammation has been recognized as a major factor involved in obesity progression, given its role in the neuroendocrine regulation of hunger, satiety, and energy expenditure. This central inflammatory response suggests that air pollution can interfere with neuroendocrine control of metabolism, favoring positive energy balance and fat accumulation independent of caloric intake alone.

Similar neuroendocrine disturbances have been described in OSA, where chronic intermittent hypoxia and sleep fragmentation can disrupt hormonal pathways involved in appetite regulation and energy homeostasis. Alterations in leptin and ghrelin signaling stimulate energy intake, heighten preference for energy-dense foods rich in fat and refined carbohydrates, and promote obesity progression in individuals with OSA (Gileles-Hillel et al., 2016; Ciriello et al., 2022; He et al., 2022; Öztürk et al., 2022).

### Gut dysbiosis

Alterations in gut microbiota composition represent another common process connecting PM_2.5_ exposure and OSA to obesity development. Both conditions have been linked to intestinal dysbiosis, defined by reduced microbial diversity, altered bacterial composition, and impairment of gut barrier integrity (Guo et al., 2025; Yu et al., 2025). PM_2.5_ itself may contain LPS, which can contribute directly to systemic inflammatory responses after entering circulation (Chen et al., 2021, 2022).

Although experimental findings suggest that PM_2.5_ may alter gut microbial composition, direct evidence in human populations remains limited. Experimental studies demonstrated that air pollution exposure could substantially alter intestinal homeostasis. PM_2.5_ has been associated with reduced abundance of beneficial microbial taxa, altered production of microbial metabolites, disruption of epithelial barrier integrity, and increased intestinal oxidative stress and inflammation (Li et al., 2022; Yu et al., 2025). These alterations might favor bacterial translocation and endotoxemia, leading to systemic metabolic dysfunction beyond the respiratory tract (Zermeño-Ruiz et al., 2026). Similarly, OSA is related to gut microbiota alterations and impairment of intestinal barrier function (Guo et al., 2025; Karthik et al., 2026). Recent data point toward the possibility that intermittent hypoxia might reduce microbial diversity and promote shifts in bacterial populations involved in inflammatory and metabolic pathways, while compromising intestinal barrier integrity. These alterations can favor metabolic endotoxemia and systemic inflammation observed in individuals with OSA (Guo et al., 2025; Karthik et al., 2026).

Intestinal microbiota plays a significant role in host metabolism and energy homeostasis. Evidence suggests that obesity is connected to alterations in gut microbial composition and diversity, potentially affecting nutrient absorption, energy harvest, and storage. Gut dysbiosis has been linked to changes in microbial metabolites, including short-chain fatty acids (SCFAs). Higher SCFA production has been found in both humans and animal models with obesity, contributing to greater caloric availability to the host. In parallel, the gut microbiome of individuals with obesity appears to harbor a greater abundance of catabolic genes, which may enhance dietary energy extraction and SCFA generation (Breton et al., 2022).

These microbial alterations are frequently accompanied by impairment of intestinal barrier integrity. Disruption of intestinal permeability facilitates translocation of bacterial components, including LPS, into systemic circulation, promoting metabolic endotoxemia and chronic low-grade inflammation (Di Vincenzo et al., 2024). Elevated circulating LPS concentrations are associated with insulin resistance and metabolic disturbances involved in obesity pathophysiology (Trøseid et al., 2013). These mechanisms might contribute to insulin resistance, adipose tissue expansion, and obesity development (Breton et al., 2022).

An additional, yet insufficiently explored, pathway may involve the incretin system. Because PM_2.5_ exposure has been related to gut dysbiosis, altered microbial metabolites, intestinal inflammation, and impaired barrier integrity, it is biologically plausible that particulate exposure could influence endogenous glucagon-like peptide-1 (GLP-1) secretion or signaling through microbiota-enteroendocrine interactions (Yu et al., 2025; Angelini et al., 2024; Shao et al., 2024). Although these observations support the biological plausibility of an interaction between PM_2.5_ exposure, gut microbiota alterations, and incretin signaling, direct evidence demonstrating that PM_2.5_ impairs GLP-1 secretion or action in humans remains lacking. Therefore, this pathway should currently be considered a promising hypothesis that warrants further mechanistic and clinical investigation.

These findings support a mechanistic link between PM_2.5_ exposure and OSA through gut-mediated inflammatory responses that may contribute to obesity development and metabolic impairment.

## Discussion

Recent findings connecting PM_2.5_ exposure, OSA, and obesity emphasize the complexity of obesity pathophysiology and reinforce the need to consider environmental and sleep-related factors alongside traditional behavioral determinants. Epidemiological studies consistently associate chronic PM_2.5_ exposure with higher adiposity, metabolic dysfunction, and increased OSA burden (He et al., 2026; Luo et al., 2024; Munir et al., 2026). Mechanistic findings suggest that both air pollution and OSA can promote obesity through convergent pathways involving systemic inflammation, oxidative stress, neuroendocrine dysregulation, and intestinal dysbiosis (Lee, 2025; Heffernan et al., 2024).

Recognition of air pollution as a factor involved in metabolic disruption can help broaden current perspectives on obesity beyond caloric imbalance alone. Experimental studies indicated that PM_2.5_ exposure can induce inflammatory activation, oxidative stress, hypothalamic dysfunction, and leptin resistance (Zhang et al., 2024; Campolim et al., 2020). Likewise, intermittent hypoxia and sleep fragmentation have been implicated in sympathetic activation, inflammatory signaling, hormonal imbalance, and metabolic impairment (Kheirandish-Gozal and Gozal, 2019; Gileles-Hillel et al., 2016). These mechanisms could lead not only to obesity onset, but also to difficulty maintaining long-term metabolic control.

Sleep duration and sleep quality may represent important mediators linking air pollution exposure to obesity. Beyond its association with OSA, chronic exposure to PM_2.5_ has been associated with increased sleep duration and poorer sleep quality, all of which have been independently associated with adverse metabolic outcomes and weight gain (Yuan et al., 2024; Knowlden et al., 2024). The extent to which sleep disturbances mediate the relationship between PM_2.5_ exposure and obesity remains poorly understood, representing an important area for future longitudinal studies.

Gut dysbiosis represents another potential pathway linking PM_2.5_ exposure, OSA, and obesity, although the strength of the available evidence differs across these associations. While alterations in gut microbial composition have been consistently reported in individuals with obesity and OSA (Guo et al., 2025; Yu et al., 2025), evidence directly connecting PM_2.5_-induced dysbiosis to metabolic dysfunction in humans remains limited. Most mechanistic data originate from preclinical studies, highlighting the need for well-designed longitudinal studies integrating environmental exposure assessment, gut microbiota profiling, and sleep phenotyping. These findings identify intestinal dysbiosis as a potential shared biological pathway through which PM_2.5_ exposure and OSA may contribute to metabolic dysfunction.

The strength of evidence differs across the proposed relationships. The role of obesity as a major risk factor for OSA is well established, whereas the independent contribution of OSA to weight gain and the influence of PM_2.5_ on obesity and OSA remain areas of active investigation. Although epidemiological and experimental findings support these associations, the available literature does not provide the same level of certainty across all proposed pathways.

Several limitations should therefore be considered when interpreting current findings. Most available research is observational, limiting causal inference, and considerable heterogeneity exists regarding pollutant composition and exposure assessment methods. OSA remains substantially underdiagnosed in some populations (Ravesloot et al., 2012; Romarheim et al., 2025), despite its high prevalence (Tufik et al., 2026), which may underestimate the magnitude of its interaction with environmental exposures. Mechanistic data are largely derived from experimental and animal studies, emphasizing the need for translational investigations in human populations. Because this article is a narrative review, it is inherently subject to selection bias. Although the literature search was designed to capture the most relevant evidence, studies were selected based on their relevance to the proposed scope rather than through a predefined systematic review protocol or formal risk-of-bias assessment.

Potential confounders and effect modifiers should be considered when interpreting current findings. Socioeconomic status, traffic-related noise, ambient temperature, smoking, dietary patterns, physical activity, urbanization, access to green spaces, and differences between indoor and outdoor pollutant exposure may influence both metabolic and sleep outcomes. The chemical composition and emission sources of PM_2.5_ can vary substantially across regions (Thangavel et al., 2022), potentially affecting biological responses beyond particle mass concentration alone.

Another important consideration is the potential role of environmental noise pollution, which frequently co-occurs with traffic-related air pollution. Noise exposure has independently been associated with sleep disruption, circadian misalignment, stress activation, and metabolic impairment (Arregi et al., 2024). Future epidemiological studies should account for both air and noise pollution to better disentangle their independent and combined contributions to obesity and sleep disorders.

Environmental health inequities provide an important framework for understanding the relationship between PM_2.5_ exposure, OSA, and obesity. Individuals living in socioeconomically disadvantaged communities are often disproportionately exposed to higher levels of air pollution due to residential proximity to major roadways, industrial areas, and other emission sources. At the same time, these populations frequently experience reduced access to healthy food, safe environments for physical activity, preventive healthcare, and specialized sleep medicine services, factors that may increase obesity risk and contribute to the underdiagnosis and undertreatment of OSA (Fuller et al., 2022; Peng et al., 2025). These overlapping environmental and social determinants may amplify metabolic vulnerability and contribute to an unequal distribution of disease burden (Alvarenga et al., 2026). Consequently, future studies should incorporate socioeconomic indicators to better characterize these interactions, while public health strategies should address both environmental exposures and social inequities to reduce the burden of obesity and sleep disorders.

Despite these limitations, the findings have relevant public health implications, mainly in densely urbanized regions where air pollution, obesity, and OSA frequently coexist. The convergence of environmental exposures with traditional metabolic and behavioral risk factors supports a more integrated approach to disease prevention and management (Alvarenga et al., 2026; Zhu and Lv, 2025). Incorporating environmental exposure assessment into obesity and sleep health research may improve risk stratification, facilitate the identification of vulnerable populations, and inform public health strategies aimed at reducing the combined burden of metabolic and sleep disorders.

Future longitudinal studies simultaneously quantifying PM_2.5_ exposure, objective sleep parameters, body composition, inflammatory biomarkers, and gut microbiota profiles are needed to determine whether these factors interact synergistically in obesity development. Further investigation of inflammatory signaling, oxidative stress, neuroendocrine alterations, and gut microbiota dysfunction could help identify novel therapeutic targets and preventive strategies. A better understanding of these interconnected mechanisms may help inform more comprehensive approaches for obesity management, particularly in urban settings where environmental and sleep-associated stressors are continuously present.

### Conceptual framework

The evidence reviewed in this article supports a conceptual framework in which PM_2.5_ exposure, OSA, and obesity should not necessarily be viewed as independent conditions, but as factors that may progressively reinforce one another. Obesity increases the likelihood of developing OSA, whereas OSA contributes to inflammatory, neuroendocrine, and metabolic disturbances that may favor further weight gain. Chronic PM_2.5_ exposure activates many of these same biological pathways, potentially amplifying metabolic dysfunction. Although current data does not establish whether these interactions are synergistic or merely additive, the convergence of epidemiological observations and shared mechanisms suggests that individuals simultaneously exposed to elevated PM_2.5_ levels and OSA could experience greater metabolic vulnerability than expected from either condition alone. Longitudinal studies specifically designed to investigate these interactions remain necessary.

### Strengths and limitations of the current evidence

The level of evidence supporting the proposed relationships differs substantially across the pathways discussed in this review. The association between obesity and OSA is well established and supported by large epidemiological studies, meta-analyses, and well-described physiological mechanisms. In contrast, even though recent research links long-term PM_2.5_ exposure with obesity and OSA, these associations are derived predominantly from observational studies, making causal inference challenging due to potential residual confounding related to socioeconomic status, lifestyle, urbanization, physical activity, diet, smoking, and co-exposure to other environmental factors. The magnitude of the reported associations is generally modest and considerable heterogeneity exists regarding pollutant composition, exposure assessment, and study populations.

Mechanistic findings also vary in strength. Experimental studies consistently demonstrate that PM_2.5_ can induce systemic inflammation, oxidative stress, hypothalamic dysfunction, and metabolic alterations. Many of these findings originate from animal or cellular models using controlled exposure conditions that may not fully reproduce chronic human exposure. The translational relevance of these mechanisms requires further confirmation in well-designed human studies.

Although alterations in gut microbiota have been consistently described in individuals with OSA, evidence directly linking PM_2.5_ exposure to gut dysbiosis in humans remains limited. Current knowledge is based largely on experimental models and a small number of human observational studies, making this pathway biologically plausible, but still preliminary. Longitudinal studies integrating environmental exposure assessment, microbiome profiling, and metabolic outcomes are needed to clarify the contribution of gut dysbiosis to the relationship between PM_2.5_ exposure, OSA, and obesity.

## Conclusions

Current data suggests that PM_2.5_ exposure may contribute not only to respiratory and cardiovascular disease, but also to metabolic dysfunction and sleep disturbances linked to obesity. The overlap between air pollution exposure and OSA is highly relevant, as both conditions appear to share biological processes involved in obesity development, including systemic inflammation, oxidative stress, neuroendocrine dysregulation, and gut microbiota alterations.

These findings highlight the importance of incorporating environmental exposures into obesity and sleep health frameworks, especially in urban populations chronically exposed to elevated pollutant levels. Public health strategies aimed at reducing PM_2.5_ exposure, improving urban environmental conditions, and expanding the recognition and treatment of OSA could support metabolic health promotion at the population level.

An integrated approach considering environmental, metabolic, and sleep-related factors might help improve prevention and management strategies for obesity and its associated complications.
